# National adverse event reporting for medical devices in Israel, 2013–2024

**DOI:** 10.1186/s13584-026-00776-x

**Published:** 2026-08-24

**Authors:** Meital Mishali, Nadav Sheffer, Maya Negev

**Affiliations:** 1 School of Public Health, University of Haifa, Haifa, Israel; 2 School of Medical Engineering, Afeka—The Academic College of Engineering, Tel Aviv, Israel

**Keywords:** Medical device, Post market surveillance, Field safety notices, National reporting database, Adverse event reporting, Reporting systems, Patient safety, Israel

## Abstract

**Background:**

Post-market surveillance systems are essential for identifying medical device–related adverse events and ensuring patient safety. In Israel, the Ministry of Health has operated a national medical device reporting system for decades. The system is modeled on United States and European regulatory frameworks, although the Medical Devices Act has not yet entered into force.

**Methods:**

Following formal approval, anonymized aggregate data were obtained from the Ministry of Health Medical Device Division for reports submitted between 2013 and 2024. The dataset included annual reporting volumes, report classification as adverse events or Field Safety Notices, reporting source, and event type (death, injury, or malfunction), where available. Availability varied across the study period; analyses were therefore restricted to years in which the relevant variables were recorded.

**Results:**

Overall reporting volumes increased over time, from 406 in 2013 to 874 in 2024. Non-linear trends were observed, with declines in 2017, 2020, 2022, and 2024. Since 2019, adverse events accounted for approximately 61.9–75.5% of reports annually, while Field Safety Notices comprised 21.4–33.3%. From 2022 onward, most adverse events were classified as injuries, increasing from 87.1% in 2022 to 89.8% in 2023 and remaining high at 89.1% in 2024. Malfunctions accounted for 10.3% of adverse events in 2022, 7.9% in 2023, and 8.5% in 2024, whereas deaths remained relatively stable at 2.2–2.5%. Reporting was dominated by registration holders (70–80%), primarily importers and distributors, followed by manufacturers, with consistently limited contributions from healthcare institutions and voluntary reporters.

**Conclusions:**

This analysis of Israel’s national medical device reporting database provides an empirical baseline for assessing the structure and functioning of the country’s post-market surveillance system. Increasing reporting activity and improved event classification indicate system maturation. The predominance of reports from registration holders, alongside more limited participation by healthcare institutions and voluntary reporters, points to areas for continued development. The following measures would strengthen post-market surveillance in Israel: full implementation of the legal framework; clearer reporting responsibilities and pathways: targeted guidance and training; electronic reporting and analytical tools; improved event documentation; and structured feedback and transparency mechanisms.

## Background

Medical devices play a critical role in modern healthcare [1], yet their use can occasionally lead to adverse events, including injury or death. While pre-market clinical trials are essential for establishing initial safety and performance, they are inherently limited in scope, enrolling relatively small and carefully selected populations under controlled conditions [2]. Once a device enters broader clinical use across larger and more diverse patient populations, new safety signals may emerge that were not detectable during the pre-market phase [3]. Post-market surveillance systems are therefore essential for bridging this gap by detecting such signals and supporting the timely identification and management of emerging risks. Adverse event reporting enables the documentation and investigation of incidents that have occurred, while manufacturer-initiated corrective actions—often communicated through Field Safety Notices (FSNs)—serve to inform stakeholders of identified risks and recommended mitigation measures. Together, these mechanisms form a cornerstone of post-market surveillance, helping regulatory authorities monitor device performance, safeguard patient safety, and implement measures such as recalls, usage restrictions, or design modifications when necessary.

Many countries have established formal adverse event reporting systems to facilitate this process. In the United States, for example, manufacturers, distributors, and user facilities are legally required to report adverse events [4], and these reports are made publicly available through the FDA’s Manufacturer and User Facility Device Experience (MAUDE) database [5]. In MAUDE, however, the vast majority of reports originate from manufacturers, while contributions from voluntary reporters and other sources remain minimal [6]—a pattern that can limit the diversity of perspectives captured and potentially affect the comprehensiveness of post-market surveillance [7]. In Europe, the European Database on Medical Devices (EUDAMED) platform was established to support centralized medical device vigilance and post-market surveillance [8]; however, unlike the U.S. MAUDE database, it does not currently provide openly accessible, case-level adverse event data for independent research [9]. Despite these regulatory frameworks and reporting infrastructures, underreporting of medical device–related adverse events remains a well-documented challenge across post-market surveillance systems worldwide, as noted in multiple studies examining reporting practices and data completeness [10–12].

Israel represents an instructive case for international audiences. Despite the fact that its Medical Devices Act has not yet come into force, the country has developed post-market surveillance practices drawing on U.S. and European models. This experience illustrates how smaller jurisdictions can adapt global standards while operating within an incomplete legal framework. The Ministry of Health (MoH) has operated a medical device post-market surveillance system since the late 1990s. A significant regulatory milestone occurred in 2012, when the Medical Devices Act was passed in the Knesset (Israel’s Parliament), followed by the publication of its first regulations in 2013 [13]. Although the Act has not yet entered into force, as its implementing regulations remain under development, medical devices in Israel are subject to stringent regulatory requirements. Manufacturers and registration holders must submit a declaration upon product registration committing to report adverse events, manufacturer notices, and regulatory actions related to their devices. In practice, reporting by manufacturers and registration holders operates through licensing conditions and Ministry of Health guidance, rather than through a fully implemented statutory vigilance framework. By contrast, healthcare institutions report incidents mainly through a general patient-safety procedure managed by the Medical Administration, that is not specific to medical devices [14], although some device-related events are also reported directly and voluntarily to the Medical Device Division. Over the years, the MoH Medical Device Division has been developing and refining its reporting database, in line with practices adopted by major regulatory authorities such as the FDA, to improve the system’s capacity to capture, organize, and analyze safety-related information. Unlike in some jurisdictions, Israel’s adverse event and FSN reporting database is not publicly accessible, and detailed analyses of its content have not been available to date. The lack of published analyses has limited academic insight into the system’s structure, trends, and international comparability. The present study seeks to address this gap by providing an analysis of the Israeli MoH’s national medical device reporting database. Covering the years 2013–2024, it examines trends in reporting volume, the distribution between adverse events and FSNs, the primary sources of reports, and the classification of events by type where available. By comparing trends and data characteristics, the study highlights the strengths and limitations of Israel’s reporting system and considers potential enhancements, including the adoption of selected elements from established systems such as the MAUDE database.

## Methods

Data access was granted following a formal request to the MoH. The dataset included the annual number of reports submitted between 2013 and 2024, their source, classification (adverse event or FSN), and event type (death, injury, or malfunction). The year 2013 was selected as the starting point, although a medical device post-market surveillance system had been in place at the Ministry since the late 1990s. This year followed the passage of the Medical Devices Act in 2012 and the publication of its first implementing regulations in 2013. The database is not publicly accessible and was provided specifically for this analysis. Data availability varied across the study period, reflecting the database’s gradual expansion and refinement over time. The distinction between adverse events and FSNs was introduced only in 2019. These variations do not affect the validity of the analysis, as each indicator was examined only for the years in which it was available. Reporting sources have been recorded since 2017, while detailed classifications of event type (death, injury, or malfunction) have been available only since 2022. Reporting sources include registration holders (primarily importers/distributors), manufacturers (a category that includes both domestic and foreign), other Ministry of Health units, and voluntary reporters. Reports submitted by other Ministry of Health units typically originate from healthcare institutions.

The data were organized and analyzed using Microsoft Excel 365. For each year, the total number of submitted reports was calculated, distinguishing between adverse events and FSNs where applicable. Reporting sources were categorized and counted. Within the adverse event category, cases were further classified by event type (death, injury, or malfunction) for the years in which this information was available.

## Results

**Fig. 1:**
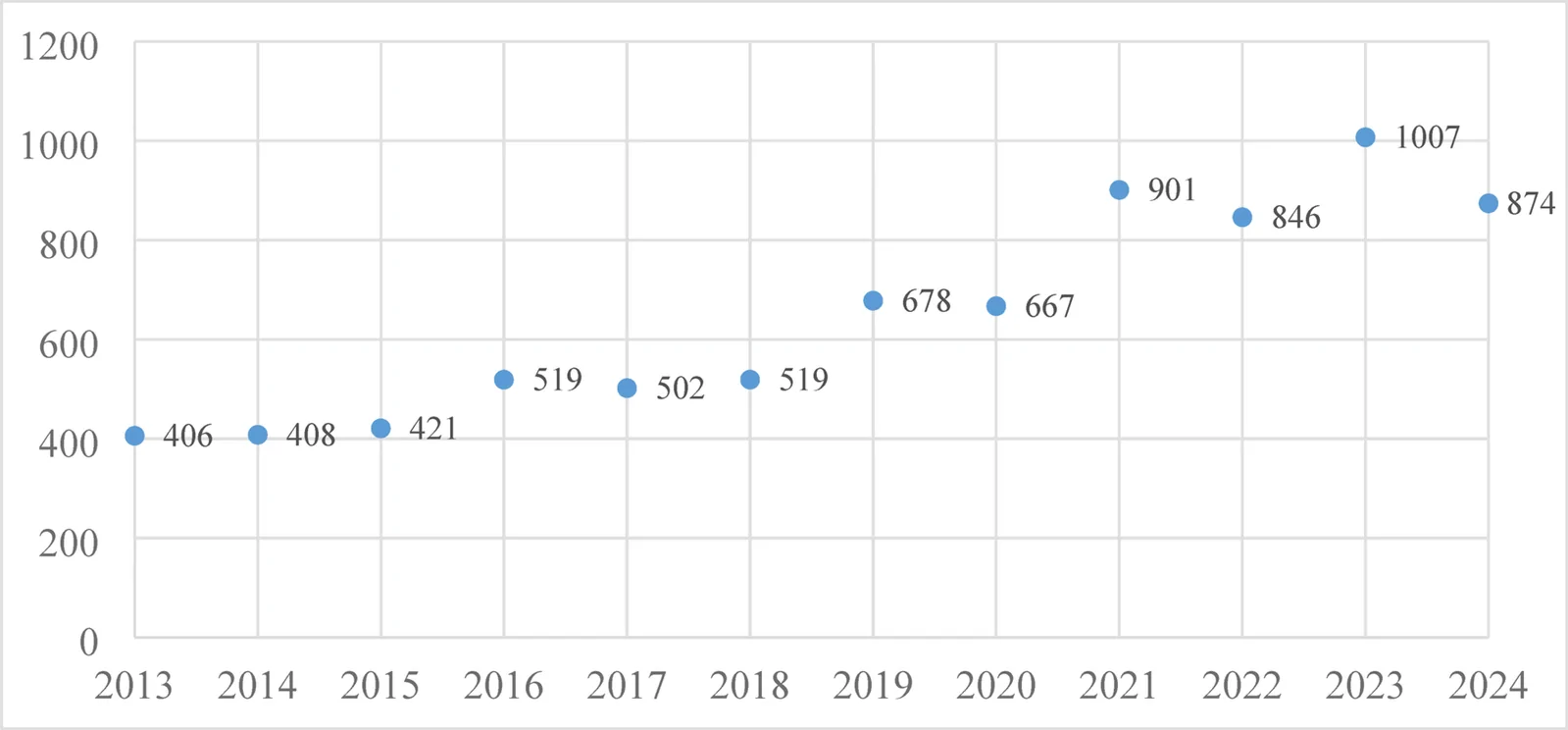
Annual number of reports received by the Medical Device Division of the Ministry of Health, 2013–2024

Figure 1 presents the annual number of reports submitted to the Medical Device Division of the MoH between 2013 and 2024. The data include both FSNs and adverse event reports. Overall, the annual number of reports increased over the study period, although the pattern was not linear. Notably, decreases in reporting were observed in 2017, 2020, 2022, and 2024 compared with the preceding year.

**Fig. 2:**
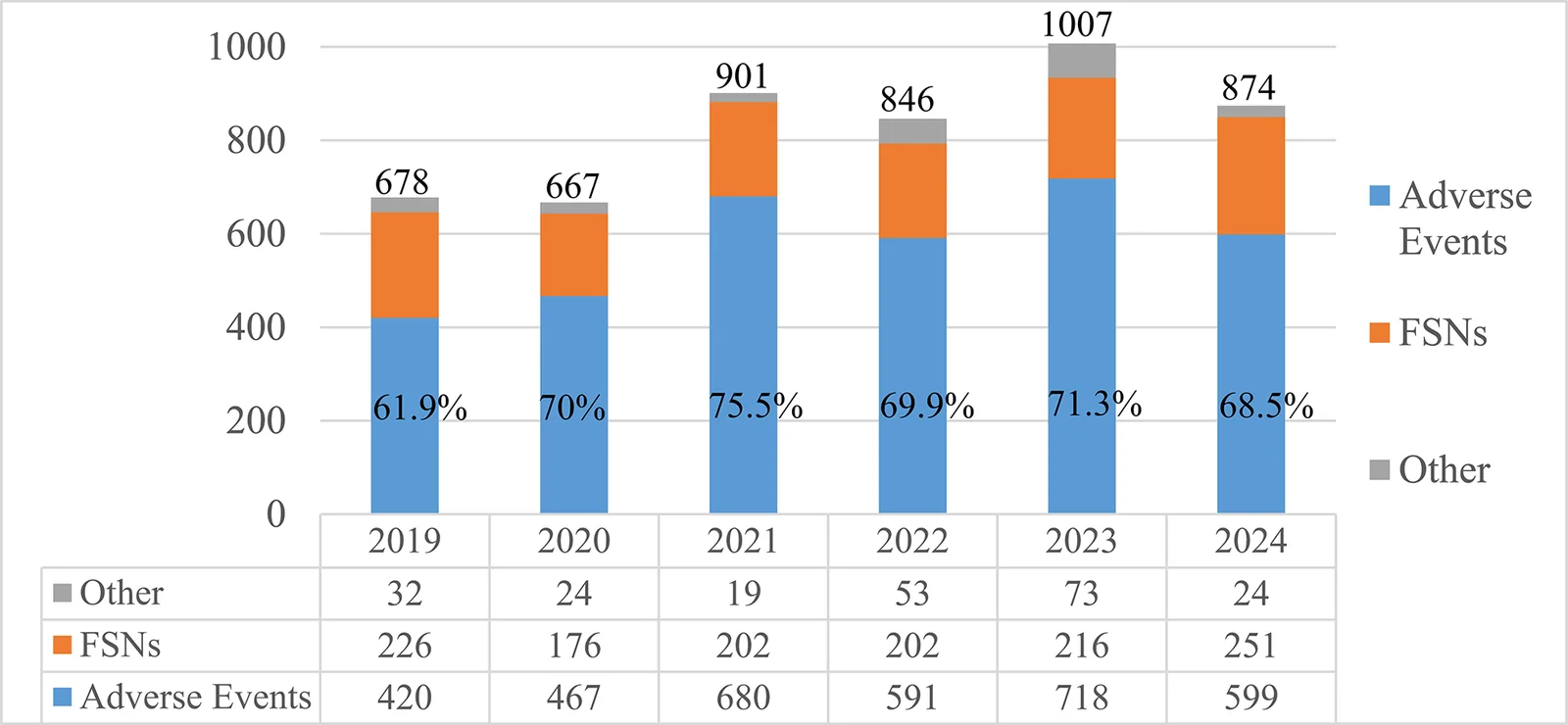
Distribution of reports between Field Safety Notices (FSNs) and adverse events for medical devices in Israel, 2019–2024

Since 2019, reports have been classified as adverse events, manufacturers’ FSNs, or other report types, as shown in Fig. 2. “Other” refers to additional safety-related reports or inquiries recorded in the database that were not classified as adverse events or manufacturers’ FSNs. Since 2019, adverse events have constituted the majority of reports each year, accounting for roughly 61.9–75.5% of all reports. Starting in 2022, adverse events were categorized into three types—death, injury, and malfunction— using categories aligned with those used in the MAUDE database (Table 1). The majority of reports were related to injuries, accounting for 87.1% of events in 2022, 89.8% in 2023, and 89.1% in 2024. Malfunctions represented a smaller share, comprising 10.3%, 7.9%, and 8.5%, respectively. Deaths were the least frequently reported, accounting for 2.2–2.5% of events across these years.

**Table 1:** Distribution of adverse events in medical devices reported in Israel (2022–2024) by event type: death, injury, and malfunction

	Death	Injury	Malfunction
2022	2.5%	87.1%	10.3%
2023	2.2%	89.8%	7.9%
2024	2.3%	89.1%	8.5%

As in the FDA system, reports originate from multiple sources. In Israel, most reports are submitted by registration holders (primarily importers/distributors), followed by manufacturers, reports relayed by other MoH divisions—typically originating from healthcare institutions—and, to a lesser extent, voluntary reporters (Table 2).

**Table 2:** Distribution of reporting sources for adverse events and Field Safety Notices (FSNs) in medical devices in Israel, 2017–2024

	Registration holders	Manufacturers	Ministry of Health divisions	Voluntary reporters
2017	80.3%	9.2%	8.6%	1.6%
2018	76%	13%	7.7%	3.1%
2019	74.3%	14.8%	6.3%	3.8%
2020	76.7%	15.6%	3.3%	3.9%
2021	70.2%	23%	4.9%	1.9%
2022	74.8%	14.6%	7.5%	1.8%
2023	80.5%	10.4%	7.7%	1.5%
2024	80.5%	12.1%	5%	2.4%

Together, these findings describe the volume, distribution, and sources of medical device adverse event and FSN reports submitted to the Ministry of Health between 2013 and 2024.

## Discussion

This analysis of Israel’s medical device adverse event and FSN reporting data from 2013 to 2024 reveals a general upward trend in reporting volumes, albeit with fluctuations in certain years. Since their distinction from FSNs in 2019, adverse events have consistently accounted for the majority of reports, with injury being the most frequently reported event type in 2022–2024. Reporting was dominated by registration holders representing manufacturers, with minimal contributions from medical institutions and voluntary reporters — a pattern that aligns with previous findings from the U.S. MAUDE database, where manufacturers likewise accounted for most reports, and healthcare facilities and voluntary reporters contributed only a small fraction of submissions [7]. These patterns provide an initial baseline for assessing the current state of post-market surveillance in Israel and serve as a starting point for discussing potential areas for system improvement.

The overall upward trend in reporting volumes from 2013 to 2024 likely reflects multiple factors, including the progressive maturation of reporting practices over time, increased awareness among stakeholders, and the expanding availability and use of medical devices in clinical practice, consistent with upward trends observed in comparable reporting systems worldwide [7, 12, 15, 16]. However, temporary decreases were observed in 2017, 2020, 2022, and 2024. The declines in 2017 and 2022 warrant further investigation, as the available data do not allow clear determination of the factors underlying these fluctuations. By contrast, the 2020 decline may be attributed to the COVID-19 pandemic’s impact on healthcare systems, which disrupted normal reporting patterns globally [17–20]. A temporary decrease in U.S. summary reports and events was also observed in 2020, supporting the possibility that pandemic-related disruptions affected reporting activity across settings [7]. Similarly, the 2024 decrease may reflect strain on Israel’s healthcare system following the war that began in October 2023, including reduced elective procedures and constrained reporting capacity, consistent with evidence that military conflicts disrupt routine hospital operations [21].

The establishment of clear distinctions between adverse events and FSNs in 2019 represents an important step in the system’s development, aligning Israel’s approach more closely with international reporting practices. This categorization provides a clearer analytical framework to support more structured interpretation of reported safety information for regulatory purposes. The consistent predominance of adverse events over FSNs (61.9–75.5% versus 21.4–33.3%) may suggests that reported adverse events constitute a substantial share of the safety information captured by the system, rather than the database being composed primarily of manufacturers’ FSNs. The distribution of adverse events by type reveals an important pattern that warrants careful interpretation. Injuries accounted for the vast majority of adverse events in 2022–2024 (87.1–89.8%), whereas malfunctions represented a much smaller proportion (7.9–10.3%). This pattern may suggest that the reports captured in the system more often involve clinically apparent events that directly affect patient care. However, the contrast with the U.S. MAUDE database, in which malfunctions constitute the predominant event type (65.3%) [7], should be interpreted cautiously. One possible explanation is underreporting of near-miss or non-harm malfunctions in the Israeli system. Another may lie in differences in event-type definitions and classification practices across systems. In Israel, reports are derived from multiple reporting formats (e.g., the European MIR, U.S. FDA Form 3500 A, the form specified in the Israeli procedure, and other manufacturer-specific forms), which do not apply a uniform event-type classification. Instead, event type is assigned upon entry into the national database based on the report content, according to local reporting definitions and procedures. As a result, events involving temporary patient harm may be classified as injuries in the Israeli system, which could broaden the injury category relative to other reporting systems. Differences in reporter composition may also contribute to the observed pattern. In the U.S. MAUDE database, most reports are submitted by manufacturers (96.5%), whereas in Israel most reports are submitted by registration holders (70–80%). Consistent with our MAUDE analysis, reporting patterns may differ by reporter type—for example, distributors in the U.S., accounting for only 2.2% of reports, submit predominantly injuries (94.8%) [7]. These patterns may also be shaped by stakeholder perspectives [22], including differing interpretations of whether responsibility lies with the device or its use [23]. These findings highlight the importance of interpreting event-type distributions within the regulatory and reporting context of each system, rather than viewing them as directly comparable indicators, and underscore the value of greater international alignment in event-type definitions and reporting frameworks to support more meaningful cross-system comparisons.

The relatively low proportion of death reports (2.2–2.5%) should be interpreted cautiously. While this may reflect the overall distribution of reported device-related harms, fatal cases are often clinically complex, and the determination of whether a death is directly attributable to a device may not always be straightforward. This proportion is comparable to and even higher than the proportion of manufacturer-submitted death reports in the U.S. FDA MAUDE database (1.1% between 2005 and 2022) [7]. The predominance of registration holders (70–80% of reports) mirrors patterns observed in other regulatory systems but also highlights potential limitations. While manufacturer-initiated reporting is essential, overdependence on this single source may limit the breadth of safety insights [22]. The overall reporting pattern is comparable to that observed in the U.S. MAUDE database, where healthcare facilities and voluntary reporters likewise account for a limited share of reports. However, Israel shows a relatively higher contribution from both medical institutions (3.3–8.6% versus approximately 0.5%) and voluntary reporters (1.5–3.9% versus approximately 0.7%) [7].

Healthcare providers are uniquely positioned to identify and report adverse events as they occur in clinical practice. Their limited participation in the current system may be due to several factors, including unclear or fragmented reporting pathways, lack of awareness about reporting requirements, insufficient training on the reporting process, and time constraints in busy clinical environments, as identified in prior qualitative research [23]. Addressing these barriers through targeted education programs [24], streamlined reporting mechanisms [25], and integration with existing clinical documentation systems could help improve the comprehensiveness of surveillance data [26, 27]. The low rate of voluntary reporting, while consistent with patterns observed in some other countries, suggests that patients and other stakeholders may be unaware of reporting opportunities or face barriers to participation [28, 29]. This issue is particularly important as an increasing number of medical devices are used in home settings, where patients and non-professional caregivers may be primary users and the first to recognize device-related problems. Patient-reported adverse events can provide valuable perspectives that may be missed by healthcare providers or manufacturers, particularly regarding long-term outcomes, quality of life impacts, and user experience issues. Experience from other jurisdictions further supports the importance of accessible reporting pathways and targeted outreach. In the United States, the FDA’s MedWatch program provides a voluntary reporting route for healthcare professionals, patients, and consumers [30], while Regulation (EU) 2017/745 states that Member States should take appropriate measures, including targeted information measures, to encourage and enable reporting by healthcare professionals, users, and patients [31].

The adoption in 2022 of a classification scheme mirroring MAUDE’s categories, represents a further step toward international harmonization. Continued development toward more advanced database platforms, with improved analytical tools and greater transparency for researchers and healthcare professionals, could further strengthen surveillance capabilities. Established systems such as MAUDE illustrate how accessible databases can support research, clinical decision-making, and collaborative safety initiatives.

This analysis is subject to several limitations. The varying availability of data elements across the study period reflects the system’s evolution but limits the depth of longitudinal analysis. In addition, direct year-by-year comparison with U.S. reporting trends across the full study period was not feasible, although such comparison might have helped distinguish between trends associated with global influences and those related to local events. This was because the available U.S. analysis extended only through 2022, comparable categorization of adverse event reports within the Israeli database was available only for the later years of the study period, and differences in reporting structure between the systems further limited comparability. Finally, the database does not include denominator or exposure data, and observed reporting trends over time may therefore reflect not only underlying safety patterns but also changes in reporting practices, reporting awareness, and database development.

Future research should focus on longitudinal analyses of defined device categories, investigation of factors influencing reporting patterns, and comparison with international reporting systems, particularly in smaller countries with more comparable regulatory and healthcare contexts. It should also examine the potential role of AI-based tools in adverse event reporting and post-market surveillance, including their use in report generation, signal detection, and identification of events from clinical data. In parallel, the growing integration of AI into healthcare highlights the importance of considering how surveillance systems may need to adapt in the future.

## Policy recommendations

Based on the present findings, several collaborative steps may help further strengthen Israel’s post-market surveillance system. These suggestions focus on nurturing existing workflows, improving communication, and fostering data sharing at a pace that aligns with available resources and institutional priorities:

1. **Supporting Clear and Consistent Reporting Practices**: As the regulatory framework evolves, the MoH should further develop and formalize clear procedures for direct reporting to the national database by healthcare institutions, alongside reporting by registration holders. These procedures should build on existing reporting requirements and clarify reporting responsibilities, minimum data requirements, and timelines. They should also be supported by accessible electronic reporting pathways and forms appropriate to different reporter categories, including manufacturers, importers, healthcare institutions, and voluntary reporters.
2. **Enhancing Digital Tools and Infrastructure**: As resources become available, the MoH should lead the continued upgrading of current reporting and data management tools. It should introduce simpler, user-friendly electronic forms to make direct database submission more intuitive, streamline documentation, and provide MoH staff with better analytical tools for routine data reviews.
3. **Strengthening Reporting by Healthcare Institutions and Coordination**: In alignment with MoH reporting requirements and guidance, healthcare institutions should take the lead in developing clear, device-specific internal procedures, assigning reporting responsibilities, integrating reporting into existing clinical workflows, and raising awareness among clinical staff through targeted training and practical guidance. These procedures should require contemporaneous documentation of key device details, such as the exact device name, model, and serial or lot number, as well as preservation of the device and related materials following an event to enable further investigation. Healthcare institutions and registration holders should also agree on shared minimum data requirements and common terminology to streamline investigations and help reduce the recurrence of device-related incidents.

## Conclusions

Taken together, these findings highlight both the maturation of Israel’s post-market surveillance system and the structural challenges that continue to shape reporting practices. By analyzing Israel’s national medical device adverse event and FSN reporting system, this study provides empirical insight into reporting patterns, stakeholder contributions, and structural characteristics. The findings point to the system’s substantial reliance on registration holders, alongside comparatively limited participation by healthcare institutions and voluntary reporters, highlighting key features of the current system and areas for further development. As medical technologies and clinical practices continue to evolve, robust and adaptive post-market surveillance systems will remain essential for safeguarding patient safety and maintaining public trust in medical devices.

## Data Availability

The data that support the findings of this study are held by the Israeli Ministry of Health and are not publicly available due to regulatory and confidentiality restrictions. Access to these data is subject to approval by the Ministry of Health.

## Abbreviations

MAUDE: Manufacturer and user facility device experience
FDA: Food and drug administration
EUDAMED: European database on medical devices
US: United States
FSN: Field safety notice
COVID: 19–coronavirus disease 2019
MoH: Ministry of health
